# Characterization of highly pathogenic avian influenza H5Nx viruses in the ferret model

**DOI:** 10.1038/s41598-020-69535-5

**Published:** 2020-07-29

**Authors:** Joanna A. Pulit-Penaloza, Nicole Brock, Claudia Pappas, Xiangjie Sun, Jessica A. Belser, Hui Zeng, Terrence M. Tumpey, Taronna R. Maines

**Affiliations:** Influenza Division, National Center for Immunization and Respiratory Diseases, Centers for Disease Control and Prevention, MS G-16, 1600 Clifton Rd. NE, Atlanta, GA 30329 USA

**Keywords:** Microbiology, Molecular biology

## Abstract

Highly pathogenic avian influenza (HPAI) H5 viruses, of the A/goose/Guangdong/1/1996 lineage, have exhibited substantial geographic spread worldwide since the first detection of H5N1 virus in 1996. Accumulation of mutations in the HA gene has resulted in several phylogenetic clades, while reassortment with other avian influenza viruses has led to the emergence of new virus subtypes (H5Nx), notably H5N2, H5N6, and H5N8. H5Nx viruses represent a threat to both the poultry industry and human health and can cause lethal human disease following virus exposure. Here, HPAI H5N6 and H5N2 viruses (isolated between 2014 and 2017) of the 2.3.4.4 clade were assessed for their capacity to replicate in human respiratory tract cells, and to cause disease and transmit in the ferret model. All H5N6 viruses possessed increased virulence in ferrets compared to the H5N2 virus; however, pathogenicity profiles varied among the H5N6 viruses tested, from mild infection with sporadic virus dissemination beyond the respiratory tract, to severe disease with fatal outcome. Limited transmission between co-housed ferrets was observed with the H5N6 viruses but not with the H5N2 virus. In vitro evaluation of H5Nx virus replication in Calu-3 cells and the identification of mammalian adaptation markers in key genes associated with pathogenesis supports these findings.

## Introduction

Highly pathogenic avian influenza (HPAI) H5N1 viruses of the A/goose/Guangdong/1/1996 lineage were first detected in birds in China’s Guangdong province in 1996. Within a year the virus spilled over to Hong Kong poultry markets, which coincided with 18 reported human cases, 6 of which were fatal^1^. Molecular analysis of human isolates revealed a number of human adaptation markers in the PB2, PA, NP, and M genes^2^, highlighting the need for monitoring and characterization of these viruses to inform pandemic risk assessments. No additional laboratory confirmed human cases were reported until 2003, when the virus was isolated from 2 Hong Kong family members, 1 of whom died^3^. Since then, the virus evolved by drift and shift and spread throughout Asia, Europe, the Middle East, and most recently, North America. Accumulation of mutations in the HA gene of the H5N1 viruses has led to the emergence of 10 genetically distinct phylogenetic clades (0–9) and multiple subclades^4,5^ while reassortment events with other avian viruses have led to the emergence of new HPAI virus subtypes (H5Nx), including H5N2, H5N3, H5N5, H5N6, H5N8 and H5N9. Since 1997, HPAI H5N1 viruses have caused over 860 laboratory-confirmed human infections in 17 countries of which 53% were fatal^6^. Infections have occurred primarily via direct contact with infected poultry or indirect contact in live poultry markets, however limited human-to-human transmission has been reported^7^.

In early 2014, HPAI H5N8 and H5N6 viruses from a novel clade (2.3.4.4, initially designated as 2.3.4.6) drew attention because of their rapid evolution and global spread. The HPAI H5N8 virus, which was initially associated with poultry outbreaks in South Korea and shortly after in China, Japan, and Russia, spread rapidly to several European countries, reaching Canada and United States via migratory flyways by the end of the year^8^. Simultaneously, HPAI H5N6 subtype viruses from the same clade caused multiple outbreaks in China, Viet Nam, and Lao People's Democratic Republic and was associated with the first HPAI non-H5N1 virus human infection, in April 2014 in China^9,10^. Currently, H5N6 viruses have spread to multiple countries in Europe, the Middle East, and Africa^11^. A total of 24 laboratory-confirmed human cases have been reported in China that include at least 7 deaths^6^.

Considering their rapid evolution, prevalence, and capacity to cause human infection with a high mortality rate, clade 2.3.4.4 HPAI H5Nx viruses pose a serious risk to both the poultry industry and human health^12^. For these reasons, it is important to continually monitor the pathogenesis of these viruses in mammalian models to inform pandemic risk assessments. Several research groups have reported low pathogenicity, lack of extrapulmonary spread, and lack of transmission of Asian, European, and North American HPAI H5N8 and H5N2 isolates in the ferret model^13–16^. In contrast, analysis of clade 2.3.4.4 H5N6 isolates using this animal model showed enhanced pathogenicity as compared to other H5Nx viruses of the same clade. In addition, some H5N6 isolates efficiently transmitted between co-housed ferrets but none of the tested viruses was capable of transmission through air^16–19^. Notably, molecular markers related to human adaptation have been reported in the polymerase genes of some HPAI H5N6 human isolates, suggesting enhanced polymerase activity in mammalian cells^18^.

To further build upon these findings, we characterized the pathogenicity and transmissibility of three recently isolated human and avian clade 2.3.4.4 HPAI H5N6 viruses in both in vitro and in vivo models. To contextualize these viruses, we additionally evaluated a North American clade 2.3.4.4 HPAI H5N2 virus, a subtype for which previous studies have shown to cause mild disease in mammalian models in the absence of transmission^14^. All tested H5N6 viruses displayed increased pathogenicity in ferrets and replicated to significantly higher titers in vitro and in vivo, compared to the H5N2 virus; however, the H5N6 viruses exhibited pronounced strain-specific heterogeneity with regard to their capacity to cause severe and fatal disease. Transmission phenotypes of these H5 viruses also varied, further demonstrating the diversity of emerging H5Nx viruses.

## Results

### Molecular analysis and replication kinetics of H5Nx viruses in Calu-3 cells

Based on numerous studies employing ferrets, H5 subtype viruses rarely transmit in direct contact models and even less often in airborne transmission models^20^. However, a transmissible phenotype has been achieved by the introduction of mutations in the HA gene that switch the receptor binding specificity of the HA from avian- to human-like while maintaining required protein acid stability, and mutations in the PB2 gene that enable efficient replication in the mammalian respiratory tract, respectively^21–23^. As each human infection with a zoonotic strain provides an opportunity for virus adaptation to a new species, it is important to monitor the acquisition of human adaptation markers in newly emerging avian influenza strains, especially those associated with human infection. Here, we analyzed the protein sequences of 1,000 avian and 24 human H5N6 isolates available in the GISAID database (2013–2019) for the presence of key molecular markers associated with host adaptation in the HA and PB2 protein sequences (Table 1). With respect to the amino acids that play a role in HA receptor binding specificity, all analyzed isolates, including human isolates, possessed markers associated with avian receptor binding preference (224N, 226Q, and 228G; H3 numbering)^21,22^. The majority of the analyzed sequences did not possess a glycosylation motif at positions 158–160 (NXS/T), a marker associated with a transmissible phenotype^21,22^. Analysis of PB2 protein sequences revealed that unlike the avian isolates, over 50% of the analyzed human isolates possessed E627K or D701N substitutions, which have been previously linked to enhanced virus replication at the temperatures found in the human upper respiratory tract and airborne transmission in mammalian hosts^24–26^.

**Table 1:** Human adaptation markers in the HA and PB2 amino acid sequences of H5Nx viruses.

Virus	Isolate origin	Subtype	Clade^b^	HA^c^				PB2	
				158–160 NXS/T^d^	N224K	Q226L	G228S	E627K	D701N
	Human isolates (24)^a^	H5N6		12.5%	100% N	100% Q	100% G	57% K	4% N
	Avian isolates (1,000)^a^	H5N6		< 1%	100% N	100% Q	100% G	100% E	100% D
Sichuan/26221	Human	H5N6	2.3.4.4	NDA	N	Q	G	E	N
Yunnan/14563	Human	H5N6	2.3.4.4	NDA	N	Q	G	K	D
dk/Bang/19D770	Avian	H5N6	2.3.4.4	NDA	N	Q	G	E	D
tr/MN/10915	Avian	H5N2	2.3.4.4	NDA	N	Q	G	E	D
Vietnam/1203	Human	H5N1	1	NST	N	Q	G	K	D

^a^Sequences of H5N6 viruses isolated from humans (24) and birds (1,000) were downloaded from GISAID database.

^b^Clade classification was done using Influenza Research Database H5N1 Clade Classification Tool.

^c^H3 HA numbering.

^d^Glycosylation site NXS/T, where X can be any amino acid except for proline. The percentage of isolates with a potential glycosylation site is shown.

**Figure 1:**
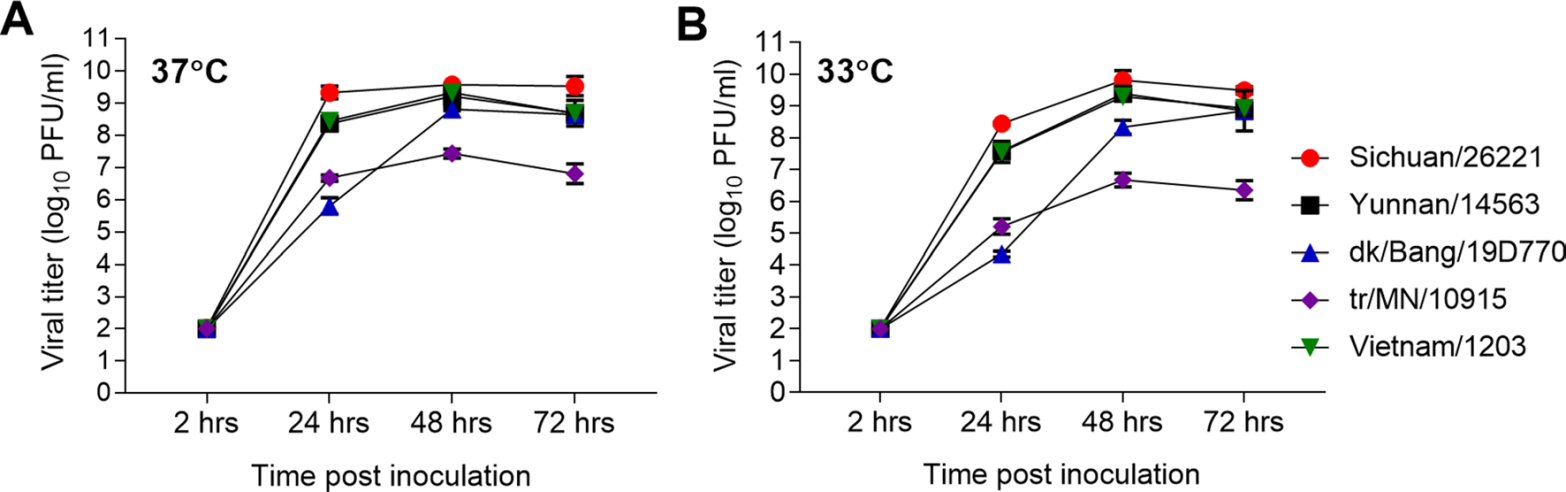
Replication kinetics of H5Nx influenza viruses in Calu-3 cells. Human airway epithelial cells (Calu-3), grown on transwell inserts, were inoculated apically in triplicate at an MOI of 0.01 with A/Sichuan/26221/2014 H5N6, A/Yunnan/14563/2015 H5N6, A/duck/Bangladesh/19D770/2017 H5N6, A/turkey/Minnesota/10915/2015 H5N2, or A/Vietnam/1203/2004 H5N1 viruses. The cells were incubated at 37 °C (**A**) or 33 °C (**B**) and culture supernatants were collected at 2, 24, 48, and 72 h p.i. for viral titer determination by standard plaque assay. Statistical significance between the titers at each time point was analyzed by two-way analysis of variance (ANOVA) with Tukey’s post-test (Supplemental Table S1).

For closer evaluation, we chose two human HPAI H5N6 isolates possessing either 627K [A/Yunnan/14563/2015 (Yunnan/14563)] or 701N [A/Sichuan/26221/2014 (Sichuan/26221)] in the PB2 protein, and two avian HPAI H5N6 [A/duck/Bangladesh/19D770/2017 (dk/Bang/19D770)] and H5N2 [A/turkey/MN/10915/2015 (tr/MN/10915)] isolates without these adaptations. We also selected a well-researched HPAI H5N1 virus [A/Vietnam/1203/2004 (Vietnam/1203)] that possesses 627K for comparison^27^. The human lung epithelial cell line, Calu-3, was employed to evaluate replication kinetics of these viruses in a representative human cell type at physiologically relevant temperatures. Cells were grown on transwell inserts leading to polarization and the formation of tight junctions emulative of the human airway epithelium^28^. Cells were inoculated at an MOI of 0.01 and incubated at temperatures representative of the lower (37 °C) and upper (33 °C) respiratory tracts of humans. Because avian influenza viruses are adapted for growth at the higher body temperatures of avian species (approximately 40–42 °C), growth at the lower temperatures of the human respiratory tract may impair the replication efficiency of influenza strains that do not possess mammalian adaptations, specifically in the polymerase proteins^25,29^. The results showed viruses that possessed E627K and/or D701N substitutions in the PB2 protein (Sichuan/26221, Yunnan/14563, and Vietnam/1203 viruses) replicated significantly better as compared to the avian isolates. Sichuan/26221 virus cultured at 37 °C displayed the highest mean titer at 24 h post-inoculation (p.i.) (9.3 log_10_ PFU/ml) as compared to other viruses (p < 0.0001; Supplementary Table S1) and peaked at 48 h p.i. with a titer of 9.6 log_10_ PFU/ml (Fig. 1A). Yunnan/14563 and Vietnam/1203 viruses replicated slightly less efficiently but overall reached comparable peak titers as the Sichuan/26221 virus at 48 h (9.2 and 9.3 log_10_ PFU/ml, respectively, p > 0.13). At 33 °C, these three viruses replicated efficiently, with a reduction in viral titer of < 1 log at 24 h compared with 37 °C (Fig. 1B). In contrast, titers of dk/Bang/19D770 H5N6 virus at 24 h p.i. were approximately 2.6 logs lower (5.8 log_10_ PFU/ml) at 37 °C and 3.2 logs lower (4.3 log_10_ EID_50_/ml) at 33 °C compared with human H5Nx isolates; however, by 48–72 h p.i. this virus reached titers on par with Yunnan/14563 and Vietnam/1203 viruses. The tr/MN/10915 virus replicated least efficiently in Calu-3 cells at either temperature (mean peak titer of 7.4 log_10_ PFU at 37 °C and 6.7 at 33 °C), significantly lower as compared to other viruses (p < 0.0001). These results show that human H5Nx virus isolates are capable of achieving higher titers more quickly than the avian isolates. Considering that human isolates acquired molecular markers for mammalian adaptation, close monitoring of these key residues is critical for early detection of H5 influenza viruses that are capable of efficient replication in human airway epithelia.

### Pathogenesis of H5Nx viruses in the ferret model

HPAI H5N1 influenza viruses have been studied extensively for their capacity to cause severe and fatal disease in the ferret model, but the pathogenicity of other H5 subtype viruses is less understood^20^. As such, we next evaluated representative H5N6 and H5N2 viruses for the ability to cause disease and spread systemically in the ferret model. Groups of 6 ferrets were intranasally inoculated with 6 log_10_ EID_50_ of virus; three were evaluated for clinical signs and virus shedding in nasal washes, while the remaining animals were humanely euthanized on day 3 p.i. for assessment of systemic spread of virus. Overall, ferrets infected by an H5Nx virus displayed 9.4–14.2% mean peak reduction of body weight and for most ferrets, fevers ranged 1.3–2.3 °C above baseline, but the severity of clinical signs and lethal outcome was quite varied (Table 2). We found that ferrets infected by either Sichuan/26221 or dk/Bang/19D770 virus presented with sneezing, nasal discharge, diarrhea, and neurological complications, ultimately leading to mortality of 100% or 66% of ferrets, respectively. In contrast, ferrets inoculated with Yunnan/14563 or tr/MN/10915 virus did not display overt respiratory clinical signs, and all recovered from infection.

**Table 2:** Summary results of pathogenesis and transmission of H5Nx viruses in ferrets.

Virus	Subtype	NW titer^a^	Weight loss (%)^b^	Temp change^c^	Leth.^d^	Nasal disch.^e^	Snz.^e^	Diarrh.^e^	Neur.^e^	Mortality^f^	DCT^g^	
											Virus detection	Seroconv
tr/MN/10915	H5N2	4.3 ± 0.2	9.4 ± 4.0	1.3 ± 0.3	1.0	0/3	0/3	0/3	0/3	0/3	0/3	0/3
Yunnan/14563	H5N6	5.5 ± 0.5	11.7 ± 2.2	1.7 ± 0.5	1.4	0/3	0/3	1/3	0/3	0/3	0/3	2/3
Sichuan/26221	H5N6	6.1 ± 0.4	14.2 ± 3.6	2.3 ± 0.5	3.4	3/3	2/3	1/3	3/3	3/3 (d3, d5, d5)	1/3*	1/2
dk/Bang/19D770	H5N6	4.1 ± 0.5	13.3 ± 6.8	2.1 ± 0.6	1.6	3/3	1/3	2/3	2/3	2/3 (d9, d10)	1/3*	0/2

^a^Average maximum nasal wash (NW) titer expressed as log_10_ EID_50_/ml ± SD of ferrets inoculated with 6 log_10_ EID_50_ of virus in 1 ml of PBS.

^b^Average maximum weight loss within 10 days of inoculation ± SD. All H5N6 virus-inoculated ferrets lost weight, while 2/3 ferrets in the H5N2 virus group displayed weight loss.

^c^Average maximum temperature increase (in °C) over the temperature recorded for each ferret on the day of inoculation (day 0) ± SD. Temperature increase above the baseline was observed for all inoculated ferrets.

^d^Relative inactivity index of ferrets (Leth) during the first 10 days post-inoculation^39^.

^e^Number of ferrets with nasal discharge (Nasal disch), sneezing (Snz), diarrhea (Diarrh), or neurological signs (Neur) over the total number of animals.

^f^Number of animals euthanized during the experiment due to neurological signs or excessive weight loss over the total number of animals. Days of euthanasia shown in parenthesis.

^g^*DCT* Direct Contact Transmission model, number of contact ferrets with detectable virus in nasal washes (virus detection) or antibodies to homologous virus in serum (seroconv) over the total number of ferrets.

*All contact ferrets with detectable virus in NWs did not survive the time course of infection; seroconversion was only tested for surviving animals.

**Figure 2:**
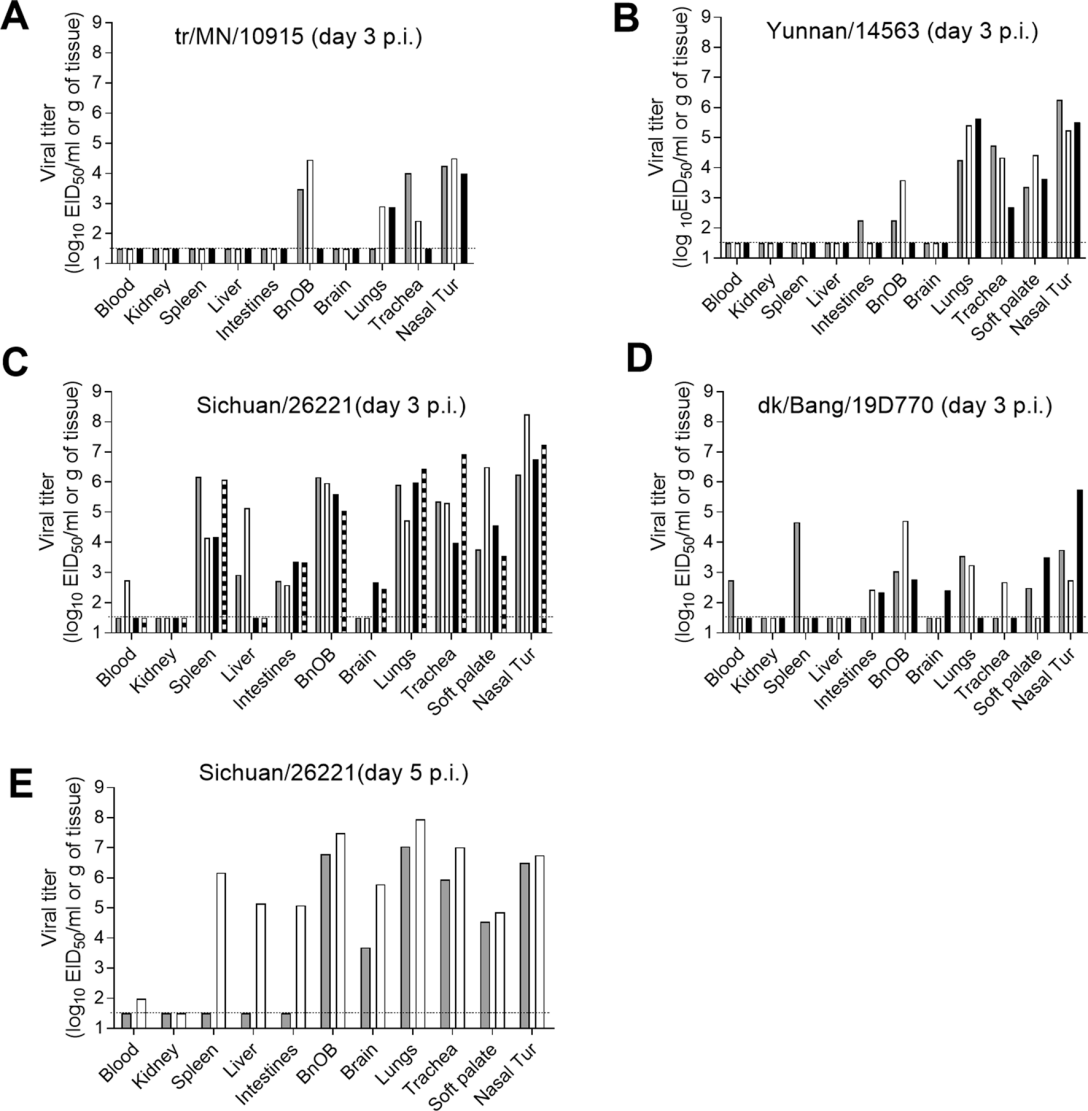
Detection of H5Nx influenza viruses in ferret tissues. Viral titers in tissues collected from ferrets 3 days post inoculation with 6.0 log_10_ EID_50_ of A/turkey/Minnesota/10915/2015 (H5N2) (**A**), A/Yunnan/14563/2015 H5N6 (**B**), A/Sichuan/26221/2014 H5N6 (**C**), A/duck/Bangladesh/19D770/2017 H5N6 (**D**) virus, or day 5 post-inoculation with A/Sichuan/26221/2014 H5N6 virus (**E**). Blood and nasal turbinate (Nasal Tur) viral titers are presented as log_10_ EID_50_/ml and kidney, spleen, liver, intestines (pooled duodenum, jejuno-ileal loop, and descending colon), olfactory bulb (BnOB), brain (pooled anterior and posterior brain), lungs (each lobe sampled and pooled), and trachea are presented as log_10_ EID_50_/g of tissue. The limit of detection is 1.5 log_10_ EID_50_/ml or g (dashed line).

All of the H5Nx viruses replicated throughout the ferret upper and lower respiratory tract, albeit with strain-specific differences. Nasal wash titers were the highest for Sichuan/26221- and Yunnan/14563-inoculated ferrets (6.1 and 5.5 log_10_ EID_50_/ml, respectively; Table 2) and on day 3 p.i., the viruses were detected in nasal turbinate, soft palate, trachea, and lung tissue from all inoculated ferrets (up to 8.3 log_10_ EID_50_/ml or g; Fig. 2). In contrast, tr/MN/10915 and dk/Bang/19D770 viruses exhibited reduced mean peak titers in nasal washes (4.1 and 4.3 log_10_ EID_50_/ml, respectively), and were recovered less often from respiratory tract tissues harvested on day 3 p.i. (titers ≤ 5.8 log_10_ EID_50_/ml or g). Extrapulmonary spread of virus at this time point was less remarkable for the two non-lethal viruses examined (tr/MN/10915 and Yunnan/14563) and was only detected in olfactory bulb and intestinal tissue, (Fig. 2A,B), which is common among influenza viruses tested in this model^27,30^. Despite the detection of tr/MN/10915 and Yunnan/14563 virus in some intestinal samples, all rectal swab samples collected on days 1, 3, and 5 p.i. tested negative for these viruses (data not shown). Dk/Bang/19D770 and Sichuan/26221 viruses, the most lethal among the group, exhibited more pervasive systemic spread of virus. Dk/Bang/19D770 virus was found in the olfactory bulb of all infected ferrets (≤ 4.7 log_10_ EID_50_/g) and less frequently in blood, spleen, intestines, and brain (Fig. 2D). Sichuan/26221 virus reached titers ≤ 6.2 log_10_ EID_50_/g in the olfactory bulb, intestine and spleen of all animals by day 3 p.i.; infectious virus was also found in the brain and liver of some animals, while viremia was noted in one animal as well. Most rectal swabs collected from dk/Bang/19D770- and Sichuan/26221-inoculated ferrets on days 2 or 3 p.i. tested positive for virus (up to 3.5 log_10_ EID_50_/ml; data not shown). Gross lesions are often observed in lung tissue collected from ferrets infected by influenza viruses, and for some highly pathogenic viruses, macroscopic manifestation of infection also can be observed in other organs^31^. In this study, macroscopic observations of tissues collected on 3 days p.i. from ferrets revealed focal and/or diffuse discoloration of 20–90% of lung tissue. The greatest coverage of lung discoloration was observed in ferrets inoculated with Sichuan/26221 or dk/Bang/19D770 virus, along with visible hemorrhages proximal to intestinal tissues and diffuse hepatic discoloration. Splenomegaly was also observed in ferrets inoculated with Sichuan/26221 virus. All Sichuan/26221 virus-inoculated ferrets were humanely euthanized on day 3 or 5 p.i. due to the development of neurological signs, as were 2/3 of the dk/Bang/19D770 virus-inoculated ferrets on day 9–10 p.i. (Table 2).

### Transmission of H5Nx viruses in the ferret model

**Figure 3:**
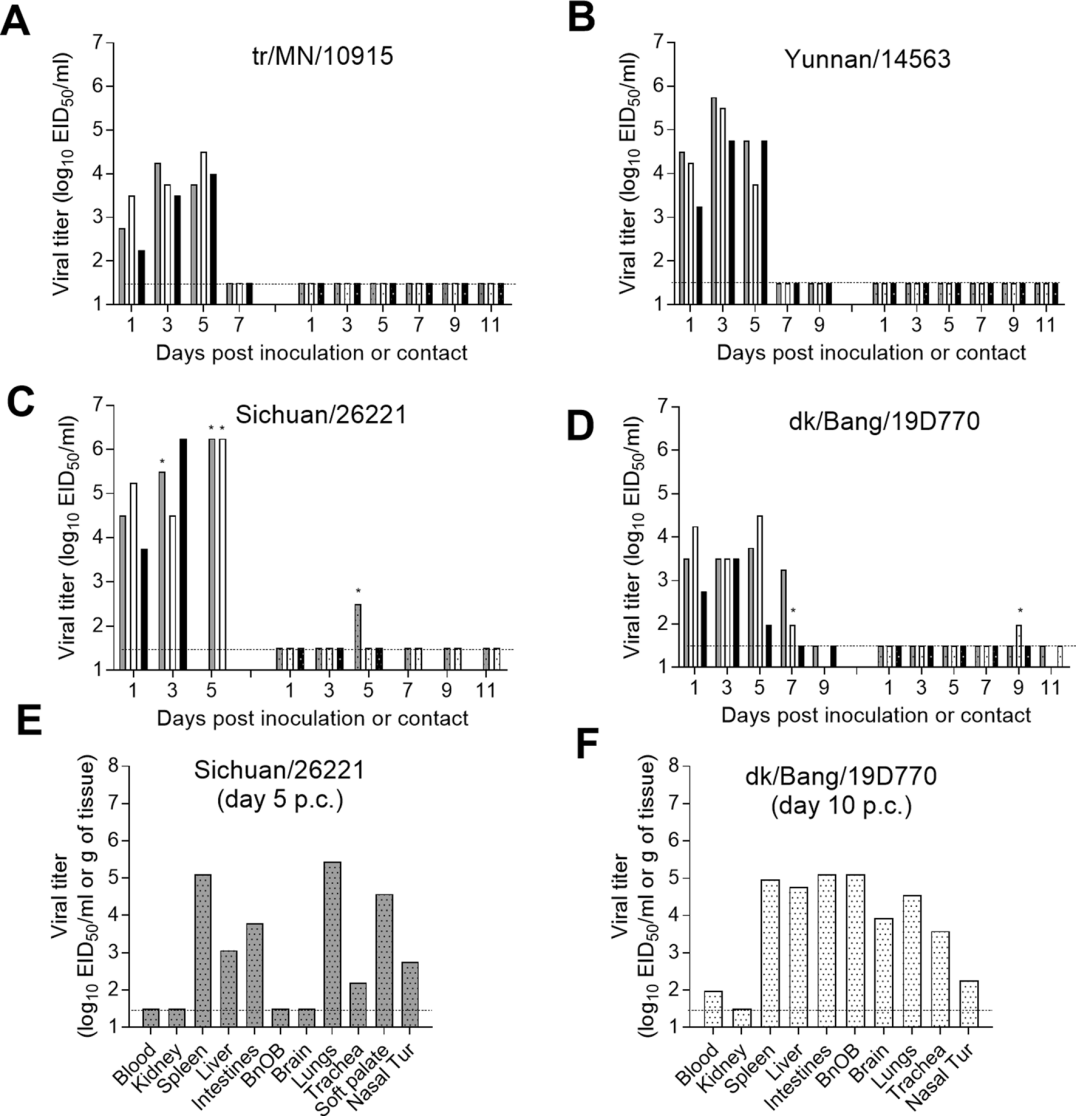
Transmission of H5Nx influenza viruses in ferrets. Three ferrets each were inoculated intranasally with 6.0 log_10_ EID_50_ of A/turkey/Minnesota/10915/2015 H5N2 (**A**), A/Yunnan/14563/2015 H5N6 (**B**), A/Sichuan/26221/2014 H5N6 (**C**), or A/duck/Bangladesh/19D770/2017 H5N6 (**D**) virus. The following day, a naïve ferret was placed in each of the three cages housing an inoculated ferret for evaluation of transmission among cohoused animal pairs. Nasal wash samples were collected from inoculated (left side of each panel) and contact ferrets (right side of each panel) every other day post inoculation or post contact and titrated in eggs. Each bar represents an individual ferret. *Indicates the last nasal wash collected before the animal was euthanized due to severe disease. Viral titers in tissues collected from contact animals are shown, A/Sichuan/26221/2014 virus (**E**), A/duck/Bangladesh/19D770/2017 virus (**F**). The limit of detection is 1.5 log_10_ EID_50_/ml or g (dashed line).

Studies describing the transmission of clade 2.3.4.4 H5Nx generally support a lack of transmission via air and a lack of or inefficient transmission in a direct contact ferret model^13,14,16,18,19^, similar to HPAI H5N1 viruses^32^. To examine if the H5Nx viruses included in the current study are capable of spreading among mammals in the presence of direct contact, 1 day after inoculation, a naïve ferret was placed in the same cage as the inoculated ferret (n = 3 per virus). The two H5Nx viruses found to cause mild disease in this model, tr/MN/10915 and Yunnan/14563 viruses, did not transmit efficiently between animal pairs as no infectious virus was detected in nasal wash specimens from any of the contact animals (Fig. 3A,B). Seroconversion to homologous virus was not observed with any of the contact animals in the tr/MN/10915 virus experiment, while 2/3 Yunnan/14563 virus contact ferrets seroconverted by the end of the study (day 23 p.c., hemagglutination inhibition titers of 640). These findings are consistent with the limited seroconversion observed in a direct contact setting with other, previously characterized, H5N1 viruses^32^.

Transmission was more evident among the animals infected by one of the more virulent viruses, Sichuan/26221 and dk/Bang/19D770. Virus was detected in 1/3 contact ferrets for both virus groups and those animals required humane euthanasia due to severe disease and neurological dysfunction on day 5 and 10 p.c., respectively (Fig. 3C,D). Interestingly, virus was not detectable in nasal washes until the day that each animal met the endpoint criteria for euthanasia. For the dk/Bang/19D770 virus-infected animal, viral titers were higher in the olfactory bulb, intestines, liver and spleen (5–6 log_10_ EID_50_/g) compared with respiratory tract tissues (≤ 4.6 log_10_ EID_50_/g), and brain tissues and blood also tested positive for infectious virus (3.9 and 2.0 log_10_ EID_50_/g or ml, respectively; Fig. 3F). Sichuan/26221 virus titers in the lungs were highest (5.4 log_10_ EID_50_/g), while spleen, liver and intestines also tested positive for infectious virus (≤ 5.1 log_10_ EID_50_/g; Fig. 3E). These data indicate that the Sichuan/26221 virus has a strong propensity for lower respiratory tract and extrapulmonary spread compared with less virulent viruses. Gross pathology revealed diffuse lung discoloration in the caudal lobes, substantial hepatic and splenic lesions, and regions of hemorrhage in intestinal and mesenteric tissues. An additional Sichuan/26221 virus contact ferret, with no detectable virus in nasal washes, seroconverted to homologous virus by the end of the study (day 21 p.c., hemagglutination inhibition titer of 640) (Table 2). Collectively, the data demonstrate a lethal outcome for any ferret that sheds detectable Sichuan/26221 virus, regardless of whether they are inoculated or become infected after exposure to inoculated animals. The high virulence of both dk/Bang/19D770 and Sichuan/26221 viruses, and capacity to cause severe and fatal disease in ferrets following virus transmission in the presence of direct contact further highlights the need to evaluate the risk that these viruses pose to public health.

## Materials and methods

### Viruses

Stocks of HPAI A/Sichuan/26221/2014 H5N6 (Sichuan/26221), A/Yunnan/14563/2015 H5N6 (Yunnan/14563), A/duck/Bangladesh/19D770/2017 (dk/Bang/19D770), A/turkey/Minnesota/10915/2015 H5N2 (tr/MN/10915), and A/Vietnam/1203/2004 H5N1 (Vietnam/1203) virus were propagated in the allantoic cavity of 10-day-old embryonated hens’ eggs at 37 °C for 24–26 h. Allantoic fluid was pooled from multiple eggs, clarified by centrifugation, aliquoted and frozen at − 80 °C. To determine the 50% egg infectious dose (EID_50_) for each virus stock, eggs were inoculated with serially diluted virus and EID_50_ was calculated using the Reed and Muench method^33^. To determine virus titers in plaque forming units (PFU), standard plaque assay in MDCK cells was performed as described previously^28^. Each stock virus was sequenced and tested for exclusivity to rule out the presence of other subtypes of influenza virus. All research with H5Nx viruses was conducted under biosafety level 3 containment, including enhancements required by the US Department of Agriculture and Select Agent Program outlined in Biosafety in Microbiological and Biomedical Laboratories^34^.

### Cell culture and viral replication

Human airway epithelial Calu-3 cells were obtained from American Type Culture Collection (ATCC; Manassas, VA). The cells were cultured on 12-well transwell plates and infected as described previously^35^. Briefly, polarized Calu-3 cells were inoculated apically in triplicate with H5Nx virus at a multiplicity of infection (MOI) of 0.01. Following a 1 h incubation period, the cells were washed three times with PBS, and then incubated at 33 °C or 37 °C in a 5% CO_2_ atmosphere. Cell culture supernatants were collected at 2, 24, 48, and 72 h p.i., and viral titers were determined by plaque assay.

### Genetic analysis

Alignments of full-length coding sequences were done using BioEdit (v7.1.3.0)^36^ and MUSCLE^37^ software. Protein identity data were obtained using MegAlign version 14.1.0 software.

### Ferret experiments

All methods performed over the course of this study were conducted in accordance with the relevant guidelines and regulations. Animal research was conducted following the ethical approval of the Centers for Disease Control and Prevention's Institutional Animal Care and Use Committee in an Association for Assessment and Accreditation of Laboratory Animal Care International-accredited animal facility. Male Fitch ferrets (Triple F Farms, Sayre, PA) 6–10 months of age were used for this study. Each animal was serologically negative for currently circulating influenza viruses as tested using standard hemagglutination inhibition assay. Ferrets were housed in Duo-Flo Bioclean mobile units (Lab Products Incorporated, Seaford, DE) during experimentation. Three ferrets per virus were intranasally inoculated with 10^6^ EID_50_ of virus diluted in PBS. For the direct contact transmission model, a serologically naive ferret was placed in the same cage as each inoculated ferret the following day^32^. This model provides the opportunity for transmission to occur by direct or indirect contact between cohoused animals, as well as by inhalation of airborne virus. Each ferret was observed daily for clinical signs of infection, including weight loss and temperature changes relative to pre-inoculation measurements. Nasal washes and rectal swabs were collected every 2 days for 2 weeks for virus titer determination. Lethargy was measured based on a scoring system used to calculate a relative inactivity index as previously described^38,39^. Three additional ferrets were inoculated as described above; on day 3 p.i., blood samples were collected using Vacutainer Heparin Tubes (BD, Mississauga Ontario) and the animals were euthanized for the assessment of systemic spread of virus^27^. Any animal that exhibited ≥ 25% weight loss or displayed neurological signs were humanely euthanized. Tissues were also collected from these animals for virus titer determination.

### Statistical analysis

Experimental results were analyzed by two-way analysis of variance (ANOVA) followed by Tukey’s post-test. Analyses were performed using GraphPad Prism 6.0 software.

## Discussion

Influenza virus pandemics occur when a novel virus, to which the general population has little or no immunity, acquires the ability to infect, cause disease, and spread among people^40^. Overall, the human population does not have protective immunity against H5 subtype viruses. Due to the rapid evolution and global prevalence of 2.3.4.4 clade H5Nx viruses, WHO has undertaken surveillance efforts to monitor the spread, emergence of reassortant viruses, and occurrence of zoonotic infections. Until 2014, HPAI H5N1 viruses were the only H5 subtype isolated from humans. The first HPAI H5N6 human infection was reported in China in April 2014 and since then, 24 human cases were reported, some of which were fatal^6^. Despite the fact that H5N1, and more recently, H5N6 subtype viruses can occasionally cause human infection, these viruses typically lack key mammalian adaptations required for sustained transmission among people^41^. In 2012, two studies demonstrated that amino acid substitutions in the HA and polymerase proteins enable HPAI H5N1 viruses to transmit through the air between ferrets^21,22^. Transmissible influenza viruses required a specific combination of 5–6 amino acid substitutions that affect polymerase activity, receptor binding specificity, and HA stability^21,22^. H5N6 subtype viruses generally maintain molecular signatures for avian-like receptor binding specificity of the HA; however, some strains have been experimentally shown to bind both avian- and human-like receptors (alpha 2,3-linked and alpha 2,6-linked sialic acids, respectively)^17,22,42,43^. More than 50% of the human isolates reported in GISAID possessed E627K or D701N substitutions in PB2 protein, markers experimentally shown to be crucial for efficient replication in mammalian cells and airborne transmission^24–26^. In this study, human HPAI H5N6 virus isolates (Yunnan/14563 and Sichuan/26221) replicated in human lung epithelial cells to comparable levels as a representative HPAI H5N1 virus (Vietnam/1203), likely attributable to high polymerase activity mediated by the presence of human adaptation markers in PB2 protein of these viruses^18^. For comparison, the avian H5N6 isolate (dk/Bang/19D770), which lacked these mammalian adaptation markers in PB2, displayed reduced replication (approximately 2.6 logs lower) at early times p.i. compared with the human isolates. Nonetheless, the H5N6 viruses reached significantly higher titers than a representative clade 2.3.4.4 HPAI H5N2 virus (tr/MN/10915) tested here and other clade 2.3.4.4 HPAI H5N2 and H5N8 viruses previously tested in this model^14^. Efficient replication of H5N6 virus in the respiratory tract of humans was also previously demonstrated using in vitro and ex vivo models. Tested H5N6 viruses replicated as well or better in ex vivo human bronchus and lung tissues compared to H5N1 and H5N8 viruses^16,43^. Identification of genetically related viruses with heterogenous virulence phenotypes in vivo underscores the need for continued assessment of molecular determinants of virulence associated with H5Nx viruses.

Viral reassortment and accumulation of molecular adaptations following infection of mammalian hosts has the potential for enhanced fitness and virulence in that host, which is why it is critical to evaluate the pathogenesis and transmission capabilities of these constantly evolving viruses in the ferret model. Previous studies show that HPAI H5N2 and H5N8 viruses generally replicated poorly throughout the respiratory tract of ferrets, while extrapulmonary spread and transmission to cohoused ferrets was rarely reported^13–16^. In contrast, reports show that H5N6 viruses replicated more efficiently in the ferret respiratory tract and were capable of occasional extrapulmonary spread, with some H5N6 strains demonstrating enhanced transmission to cage-mates, but still no airborne transmission was observed^16–19^. Consistent with these previous findings, the HPAI H5N6 viruses tested here were more pathogenic in ferrets compared to an HPAI H5N2 virus and some of the H5N6 isolates were extremely virulent and lethal to ferrets. Sichuan/26221 virus caused severe clinical signs and rapidly spread to extrapulmonary tissues and, within 5 days, was lethal to all ferrets that shed detectable virus. Extensive extrapulmonary spread and evidence of viral replication in extrapulmonary tissues was previously demonstrated for certain HPAI H5N1 virus strains using immunohistochemistry^39,44,45^. The avian isolate, dk/Bang/19D770, was highly lethal for ferrets as well, albeit delayed. In fact, replication kinetics in vitro was delayed as were the peak virus titers in ferret tissues compared with human H5N6 virus isolates. At day 3 p.i., dk/Bang/19D770 virus titers were generally lower and more sporadically detected in extrapulmonary sites. However, by 10 days p.c., dk/Bang/19D770 virus was widespread throughout the animal. This delay in peak disease and peak virus titers may be indicative of the avian isolate requiring more time than the human isolates to achieve peak levels of replication in a mammalian host. The ultimate disease and infection outcome reflects previous reports of HPAI H5N1 viruses in ferrets^27,46,47^ and humans^9,10,48^. Patients with HPAI infections presented with fever, headache, myalgia, cough, stomach discomfort, pneumonia and, in severe cases, followed by acute respiratory distress syndrome (ARDS) and septic shock. Autopsy of a deceased patient revealed virus spread outside of the respiratory tract including intestines, spleen, kidneys, and brain^10^.

In agreement with most studies of clade 2.3.4.4 H5Nx viruses in the ferret model, efficient transmission of H5N6 viruses between co-housed ferrets was not observed. Virus was detected in only 1/3 pairs following exposure to either Sichuan/26221 or dk/Bang/19D770 viruses. In both instances, the contact ferrets displayed severe signs of respiratory infection, weight loss, elevated temperature, lethargy, neurological symptoms, and were humanely euthanized. It is remarkable that virus was not detectable in nasal wash samples prior to the day that the animals required euthanasia, suggesting that virus replication was underway in the lower respiratory tract and potentially outside of the respiratory tract well before the day virus was detected in nasal washes. Perhaps this manifestation of disease is more reflective of a naturally acquired infection, as these contact animals became infected after exposure to other infected animals rather than receiving a 6 log_10_ EID_50_ intranasal inoculum. Low titers in nasal washes and nasal turbinates in conjunction with extensive extrapulmonary spread is a phenomenon previously seen with other HPAI viruses tested in this model^46,49^. As none of the viruses tested here were able to efficiently transmit among cohoused ferrets, which facilitates transmission by direct contact, indirect contact via fomites, and by inhalation of airborne virus, subsequent evaluation using a more stringent respiratory droplet transmission model, which restricts transmission events to those that are airborne, was not warranted. Our results suggest that the 2.3.4.4 clade of H5N6 viruses is not likely to spread in the human population and would need to further adapt to humans to acquire a transmissible phenotype; however, those rare infections are likely to result in severe disease. A previous study conducted in chickens showed that although acquisition of some human adaptations may have a negligible effect on virus replication, viruses containing multiple mammalian adaptation markers might be highly attenuated in birds suggesting that viruses possessing the required combination of molecular adaptations necessary for airborne transmission are not likely to emerge from birds^50^; as such, a mammalian intermediate host may be necessary for full adaptation of an avian influenza virus to humans.

Collectively, the clade 2.3.4.4 HPAI H5N6 viruses studied here displayed enhanced pathogenicity in the ferret model compared with an HPAI H5N2 virus. In some instances, the H5N6 viruses were capable of rapid progression of disease with pervasive extrapulmonary spread leading to neurological complications and ultimately death, while inefficient transmission is still a common trait. Continuous evolution of H5Nx viruses, especially those capable of human infection, highlights the need for surveillance and characterization of these viruses using in vitro and in vivo models to better inform the selection and subsequent development of candidate vaccine viruses^51^. Furthermore, additional efforts to evaluate within-host and between-host diversity of H5Nx viruses during primary infection and transmission events represent an important area of future investigation. As the clade 2.3.4.4 H5Nx viruses are currently the most prevalent to cause outbreaks in poultry^11^ and a potential source of zoonotic infections, risk assessments remain a critical component of pandemic preparedness.

## Supplementary information

Supplementary Information
