# Supplementary material for: Characterization of highly pathogenic avian influenza H5Nx viruses in the ferret model
Source: Sci Rep. 2020 Jul 29;10:12700. doi: 10.1038/s41598-020-69535-5 (PMC7391700; doi:10.1038/s41598-020-69535-5)
Supplement: Supplementary file 1 — Supplementary Information [file 41598_2020_69535_MOESM1_ESM.pdf]

Characterization of Highly Pathogenic Avian Influenza H5Nx Viruses in the Ferret Model

Joanna A. Pulit-Penaloza[1], Nicole Brock[1], Claudia Pappas[1], Xiangjie Sun[1], Jessica A. Belser[1], Hui Zeng[1],

Terrence M. Tumpey[1], Taronna R. Maines[1#]

Supplementary Table 1. Statistical analysis of replication kinetics of H5Nx influenza viruses in Calu-3 cells

	37◦C	33◦C
	24 h Adjusted P Value	
Sichuan/26221 vs. Yunnan/14563	<0.0001	0.0002
Sichuan/26221 vs. dk/Bang/19D770	<0.0001	<0.0001
Sichuan/26221 vs. Vietnam/1203	<0.0001	0.0001
Sichuan/26221 vs. tr/MN/10915	<0.0001	<0.0001
Yunnan/14563 vs. dk/Bang/19D770	<0.0001	<0.0001
Yunnan/14563 vs. Vietnam/1203	0.98	0.9998
Yunnan/14563 vs. tr/MN/10915	<0.0001	<0.0001
dk/Bang/19D770 vs. Vietnam/1203	<0.0001	<0.0001
dk/Bang/19D770 vs. tr/MN/10915	<0.0001	0.0002
Vietnam/1203 vs. tr/MN/10915	<0.0001	<0.0001
	48 h Adjusted P Value	
Sichuan/26221 vs. Yunnan/14563	0.13	0.15
Sichuan/26221 vs. dk/Bang/19D770	<0.0001	<0.0001
Sichuan/26221 vs. Vietnam/1203	0.4731	0.0493
Sichuan/26221 vs. tr/MN/10915	<0.0001	<0.0001
Yunnan/14563 vs. dk/Bang/19D770	0.0764	<0.0001
Yunnan/14563 vs. Vietnam/1203	0.9379	0.9859
Yunnan/14563 vs. tr/MN/10915	<0.0001	<0.0001
dk/Bang/19D770 vs. Vietnam/1203	0.0113	<0.0001
dk/Bang/19D770 vs. tr/MN/10915	<0.0001	<0.0001
Vietnam/1203 vs. tr/MN/10915	<0.0001	<0.0001
	72 h Adjusted P Value	
Sichuan/26221 vs. Yunnan/14563	<0.0001	0.0074
Sichuan/26221 vs. dk/Bang/19D770	<0.0001	0.0077
Sichuan/26221 vs. Vietnam/1203	<0.0001	0.0245
Sichuan/26221 vs. tr/MN/10915	<0.0001	<0.0001
Yunnan/14563 vs. dk/Bang/19D770	0.9981	>0.9999
Yunnan/14563 vs. Vietnam/1203	>0.9999	0.9913
Yunnan/14563 vs. tr/MN/10915	<0.0001	<0.0001
dk/Bang/19D770 vs. Vietnam/1203	0.999	0.9923
dk/Bang/19D770 vs. tr/MN/10915	<0.0001	<0.0001
Vietnam/1203 vs. tr/MN/10915	<0.0001	<0.0001
